# An Efficient and Facile Synthesis of 1,2,4-Aryl Triazoles and 4-Thiazolidinones Bearing 6-Fluorochroman Nucleus

**DOI:** 10.1155/2014/186207

**Published:** 2014-10-29

**Authors:** Piyush B. Vekariya, Jalpa R. Pandya, Hitendra S. Joshi

**Affiliations:** Chemical Research Laboratory, Department of Chemistry, Saurashtra University, Rajkot 360005, India

## Abstract

A new generation of chroman bearing heterocyclic five membered ring such as 1,2,4-triazoles and thiazolidinones was designed and synthesized. New chroman based nucleus 5-(6-fluorochroman-2-yl)-4-aryl-4H-1,2,4-triazole-3-thiol and 6-fluorochroman-N-(4-oxo-2-arylthiazolidinin-3-yl) chroman-2-carboxamides were synthesized. Aryl triazole compounds **4a–4j** were synthesized from 6-fluorochroman-2-carbohydrazide** 2** on reaction with base in methanol and CS_2_ followed by reaction with substituted aniline. Thiazolidinone compounds** 5a–5j** were synthesized from 6-fluorochroman-2-carbohydrazide** 2** on reaction with substituted aryl aldehyde and thioglycolic acid.

## 1. Introduction

The design and synthesis of hybrid molecules encompassing two pharmacophores in one molecular scaffold is a well-established approach to the synthesis of more potent drugs with dual activity. With this aspect, 6-fluorochroman-2-carboxylic acid derivatives in connection with thiazolidinone and 1, 2, 4-triazoles were found as a promising target for the current research project. 1, 2, 4-Triazoles and 4-thiazolidinones are the broadly investigated molecules. They have proved to be the most useful framework for biological activities among nitrogen containing five membered heterocycles. Amongst the diverse classes of heterocyclic compounds chroman, a class of oxygen containing heterocycle forms an important part of many pharmacologically active compounds. For example, the chroman ring is a constituent of various bioactive compounds that are sodium channel blocker [1], 5HT_1A_ inhibitor [2], and so forth. Commercially available antihypertensive drugs of chroman repinotan [3], robalzotan [4], and specifically 6-fluorochroman nebivolol [5] are well known. Hence, the synthesis of 6-fluorochroman derivatives is currently of significant interest in organic synthesis.

Aryl triazoles comprise various heterocyclic compounds possessing promising biological activity and are found as potential antimicrobial [6, 7] and adenosine A_2A_ receptor antagonist [8]. According to the green chemistry approach there are many solvent free reactions of 1, 2, 4-aryl triazoles that have been reported [9, 10].

4-Thiazolidinones have been widely explored for their applications in the field of medicine and agriculture [11]. They are also known as promising antimicrobial [12], antiinflammatory [13, 14], antimalerial [15], anticancer [16], tuberculostatic [17], and antiviral agents [18]. Several one-pot multicomponent syntheses of 4-thiazolidinone have been reported [19–21].

## 2. Result and Discussion

**Scheme 1:**
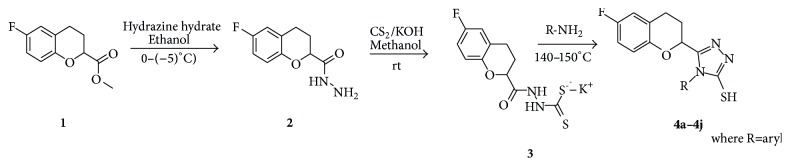
Reaction scheme for the synthesis of** 4a–4j**.

The syntheses of triazole and thiazolidinone derivatives have been previously reported by many researchers, and they normally required additional additives and long reaction time. So in this paper, we described an efficient and safe procedure for the synthesis of 4-aryl triazole containing chroman nucleus, using 6-fluorochroman-2-carboxylic acid. 6-Fluorochroman-2-carboxylic acid on esterification with methanol in the presence of concentrated H_2_SO_4_ at room temperature gave compound** 1** (Scheme 1) with good yield which on reaction with hydrazine hydrate (99%) gave compound** 2**. Compound** 2** on reaction with carbon disulphide in the presence of KOH in methanol at RT afforded compound** 3**. This on further reaction with substituted aniline without use of any solvent in fused condition yielded compounds** 4a–4j** (Scheme 1). ^1^H and ^13^C NMR spectra of the products clearly indicated the formation of triazoles** 4a–4j** in 75–95% yields (Table 1). The formation of thiol group –SH was identified by a sharp singlet at around *δ* = 11.43 ppm. By the ^13^C NMR spectrum also supported the presence of –SH group from the deshielding value of carbon attached to –SH group at *δ* 168.71 ppm.

**Scheme 2:**
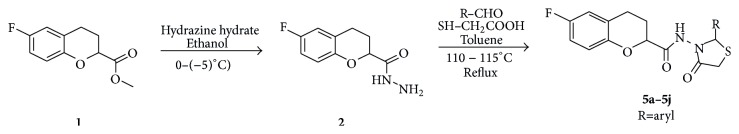
Reaction scheme for the synthesis of** 5a–5j**.

Here we introduce the one-pot synthesis of thiazolidinone from hydrazide (Scheme 2) with thioglycolic acid, substituted aryl aldehydes in toluene using a Dean-Stark assembly to synthesized compounds** 5a–5j** (Scheme 2). ^1^H and ^13^C NMR spectra of the products clearly indicated the formation of 4-thiazolidinone** 5a–5j** in 71–95% yields (Table 2). The formation of –NH group was identified by a sharp singlet at around *δ* 10.46–10.43 ppm, which is further supported by D_2_O exchange. ^13^C NMR spectrum also supported the presence of amide group from the deshielding value of carbon attached to –CONH group at *δ* 169.3 ppm and carbonyl group (part of a five member ring) at *δ* 169.47 ppm.

## 3. Conclusion

In summary, an efficient protocol for the synthesis of new 1, 2, 4-aryl triazoles and 4-thiazolidinones has been described. Herein, we are reporting the solvent free protocol for the synthesis of N-substituted 1, 2, 4-aryl triazoles** (4a–4j)** from potassium salt** (3) **. In literature, the synthesis of 4-thiazolidinones was carried out via 2 steps, but, to avoid multisteps and to make it more viable, we have developed a single-step reaction for the synthesis of 4-thiazolidinones** (5a-5j)** from hydrazide** (2) **.

## 4. Experimental

Melting points were determined in open capillary tubes and are uncorrected. Formation of the compounds was checked by TLC on silica gel-G plates of 0.5 mm thickness. IR spectra were recorded on Shimadzu FT-IR-8400 instrument using DRS (diffusive reflectance system) method. Mass spectra were recorded on Shimadzu GC-MS system (model QP-2010) using direct inlet probe technique. ^1^H NMR and ^13^C NMR were determined in CDCl_3_ and DMSO-d_6_ on a Bruker AC 400 MHz and 100 MHz spectrometer. Elemental analysis of all the synthesized compounds was carried out on Euro EA 3000 elemental analyzer and the results are in agreement with the structures assigned.

### 4.1. General Procedure for the Synthesis of Functionalized 1, 2, 4-Aryl Triazoles  **(4a–4j)**

To a stirred solution of 6-fluorochroman-2-carboxylic acid (0.01 mol) in methanol at room temperature, concentrated H_2_SO_4_ (0.01 mol) was added and reaction mixture was allowed to stir at RT for 10 hours. After completion of the reaction, solvent was evaporated and the resulting mass was poured on to ice, neutralized with saturated sodium bicarbonate solution. Separated solid precipitate was filtered, washed with water, and dried to afford methyl 6-fluorochroman-2-carboxylate** 1**.

Compound** 1** (0.01 mol) in absolute ethanol was taken into the RBF and cooled at (−5)°C. To the previously cooled solution hydrazine hydrate (99%, 0.08 mol) was added and reaction mixture was allowed to stir at 0–(−5)°C for 10 hours. After the completion of reaction separated solid residues were filtered, washed with cold ethanol, and dried to afford 6-fluorochroman-2-carbohydrazide** 2**, yield: 2.0 g (98%).

To a mixture of compound** 2** (0.1 mol) and potassium hydroxide (0.15 mol) in methanol carbon disulphide (0.15 mol) was added dropwise. Reaction mass was allowed to stir at RT for 22–24 hours. After completion of reaction the obtained solid was filtered, washed with diethyl ether, and dried to afford compound** 3**. There is no need to purify the salt for further reaction.

An equimolar mixture of potassium 2-[(6-fluorochroman-2-yl) carbonyl] hydrazine carbodithioate** 3** (0.01 mol) and substituted aniline (0.01 mol) was taken in RBF and heated at 140–150°C for 12–15 hours until the evolution of H_2_S gas ceased. After completion of reaction solid residue was dissolved in DMF, treated with dilute HCl, and poured on crushed ice. The product was isolated and crystallized from ethanol to give compounds** 4a–4j** as analytical pure product.

#### 4.1.1. 5-(6-Fluorochroman-2-yl)-4-(*p*-tolyl)-4*H*-1, 2, 4-triazole-3-thiol **(4a)**

Yield 89%, mp 170–172°C; IR (DRS): 3076(Ar, C–H str.), 2924(C–H str.), 2573(–SH str.), 1735(C=O str.), 1087(C–N str.), 1041(C–O–C str.) cm^−1^; ^1^H NMR (400 MHz, CDCl_3_): *δ* ppm 2.02–2.44(m, 5H, 2CH, 3CH), 2.65–2.96(m, 2H, 2CH), 4.83–4.90(dd, *J* = 12.6 Hz, 6 Hz, 1H, CH), 6.60–6.64(m, 1H, ArH), 6.73–6.76(m, 2H, ArH), 7.05–7.49(m, 4H, ArH), 11.43(s, 1H, SH). ^13^C NMR (100 MHz, CDCl_3_): *δ* ppm 20.3(CH_3_), 26.27(CH_2_), 28.87(CH_2_), 85.3(CH), 108.52(CH), 112.98(CH), 116.81(CH), 119.54(CH), 129.2(C), 131.16(C), 133.09(CH), 136.12(C), 139.69(C), 155.8(C), 158.29(C), 173.99(C). MS:* m/z* = 341 [M]^+^; Anal. Calcd for C_18_H_16_FN_3_OS: C, 63.32; H, 4.72; N, 12.31. Found: C, 63.23; H, 4.41; N, 12.28%.

#### 4.1.2. 4-(3-Chlorophenyl)-5-(6-fluorochroman-2-yl)-4*H*-1, 2, 4-triazole-3-thiol **(4b)**

Yield 86%, mp 205–207°C; IR (DRS): 3066(Ar, C–H str.), 2914(C–H str.), 2850(C–H str.), 2533(–SH str.), 1558(Ar, C=C bend.), 1375(C–H ben), 800(C–Cl str.), 1087(C–N str.), 1041(C–O–C str.) cm^−1^; ^1^H NMR (400 MHz, DMSO-d_6_): *δ* ppm 2.27–2.30(m, 2H, 2CH), 2.82–2.95(m, 2H, 2CH), 4.92–4.95(dd, *J* = 6.12 Hz, 12 Hz, 1H, CH), 6.50–6.54(m, 1H, ArH), 6.75–6.79(m, 2H, ArH), 7.41–7.43(m, 1H, ArH), 7.53–7.55(m, 3H, ArH), 14.0(s, 1H, SH). ^13^C NMR (100 MHz, DMSO-d_6_): *δ* ppm 25.27(CH_2_), 28.27(CH_2_), 85.3(CH), 112.98(CH), 116.81(CH), 120.08(CH), 122.3(CH), 125.68(CH), 129.2(C), 133.41(CH), 138.88(C), 139.69(C), 142.37(C), 155.8(C), 158.29(C), 173.99(C) MS:* m/z* = 361 [M]^+^; Anal. Calcd for C_17_H_13_ClFN_3_OS: C, 56.43; H, 3.62; N, 11.61. Found: C, 56.18; H, 3.49; N, 11.59%.

#### 4.1.3. 5-(6-Fluorochroman-2-yl)-4-(4-fluorophenyl)-4*H*-1, 2, 4-triazole-3-thiol **(4c)**

Yield 78%, mp 188–190°C; IR (DRS): 3030(Ar, C–H str.), 2558(–SH str.), 1581(–C=C–, str.), 1225(C–O–C str.) cm^−1^; ^1^H NMR (400 MHz, CDCl_3_): *δ* ppm 2.21–2.46(m, 2H, 2CH), 2.75–2.85(m, 2H, 2CH), 4.88–4.93(dd, *J* = 12.6 Hz, 6 Hz, 1H, CH), 6.51–6.55(m, 1H, ArH), 6.77–6.81(m, 2H, ArH), 7.53–7.57(m, 3H, ArH), 13.05(s, 1H, SH). ^13^C NMR (100 MHz, CDCl_3_): 26.27(CH_2_), 28.87(CH_2_), 85.3(CH), 108.52(CH), 112.98(CH), 116.81(CH), 118.6(CH), 122.97(CH), 129.2(C), 133.7(C), 139.69(C), 155.8(C), 158.29(C), 160.51(C), 173.99(C). MS:* m/z* = 345 [M]^+^; Anal. Calcd for C_17_H_13_F_2_N_3_OS: C, 59.12; H, 3.79; N, 12.17. Found: C, 59.02; H, 3.53; N, 12.01%.

#### 4.1.4. 4-(2, 5-Dimethylphenyl)-5-(6-fluorochroman-2-yl)-4*H*-1, 2, 4-triazole-3-thiol **(4d)**

Yield 95%, mp 123–125°C; IR (DRS): 3074(Ar, C–H str.), 2984(C–H str.), 1645(–C=C–, str.), 1468(C–H bending) cm^−1^; ^1^H NMR (400 MHz, CDCl_3_): *δ* ppm 1.92(s, 6H, 3CH, 3CH), 2.21–2.46(m, 2H, 2CH), 2.75–2.85(m, 2H, 2CH), 4.85–4.92(dd, *J* = 12.6 Hz, 6 Hz, 1H, CH), 6.72–6.95(m, 3H, ArH), 7.29–7.43(m, 3H, ArH), 13.05(s, 1H, SH). ^13^C NMR (100 MHz, CDCl_3_): *δ* ppm 17.98(CH_3_), 26.87(CH_2_), 28.87(CH_2_), 30.2(CH_3_), 85.3(CH), 108.52(CH), 116.81(CH), 118.6(CH), 112.98(CH), 124.19(CH), 132.61(CH), 134.95(C), 138.13(C), 140.75(C), 146.2(C), 155.8(C), 158.29(C), 175.05(C). MS:* m/z* = 355 [M]^+^; Anal. Calcd for C_19_H_18_FN_3_OS: C, 64.21; H, 5.10; N, 11.82. Found: C, 64.16; H, 4.93; N, 11.78%.

#### 4.1.5. 4-(3, 4-Dimethylphenyl)-5-(6-fluorochroman-2-yl)-4*H*-1, 2, 4-triazole-3-thiol **(4e)**

Yield 84%, mp 163–165°C; IR (DRS): 3081(Ar, C–H str.), 2975(C–H str.), 2575(–SH str.), 1641(C–H bending), 1579(–C=C–, str.), 1142(C–F str.) cm^−1^; ^1^H NMR (400 MHz, CDCl_3_): *δ* ppm 2.34(s, 6H, 3CH, 3CH), 2.21–2.46(m, 2H, 2CH), 2.75–2.85(m, 2H, 2CH), 4.85–4.92(dd, *J* = 12.6 Hz, 6 Hz, 1H, CH), 6.72–6.95(m, 3H, ArH), 7.29–7.43(m, 3H, ArH), 13.05(s, 1H, SH). ^13^C NMR (100 MHz, CDCl_3_): *δ* ppm 19.0(CH_3_), 20.01(CH_3_), 26.27(CH_2_), 28.87(CH_2_), 85.3(CH), 112.98(CH), 116.81(CH), 117.91(CH), 124.04(CH), 129.2(C), 132.85(C), 133.52(C), 139.69(C), 141.78(C), 143.79(C), 155.8(C), 158.29(C), 173.998(C). MS:* m/z* = 355 [M]^+^; Anal. Calcd for C_19_H_18_FN_3_OS: C, 64.21; H, 5.10; N, 11.82. Found: C, 64.09; H, 5.03; N, 11.50%.

#### 4.1.6. 5-(6-Fluorochroman-2-yl)-4-(2-fluorophenyl)-4*H*-1, 2, 4-triazole-3-thiol **(4f)**

Yield 79%, mp 108–110°C; IR (DRS): 3080(Ar, C–H str.), 2983(C–H str.), 2561(–SH str.), 1629(C–H bending), 1572(C–H bending), 1525(–C=C–, str.) 1196(C–O–C str.) cm^−1^; ^1^H NMR (400 MHz, CDCl_3_): *δ* ppm 2.34(s, 6H, 3CH, 3CH), 2.21–2.46(m, 2H, 2CH), 2.75–2.85(m, 2H, 2CH), 4.90–4.97(dd, *J* = 12.6 Hz, 6 Hz, 1H, CH), 6.72–6.95(m, 3H, ArH), 7.24–7.75(m, 3H, ArH), 13.05(s, 1H, SH). ^13^C NMR (100 MHz, CDCl_3_): *δ* ppm 26.27(CH_2_), 28.87(CH_2_), 85.3(CH), 108.52(CH), 112.98(CH), 116.81(CH), 119.7(C), 119.58(CH), 129.2(C), 129.95(CH), 130.09(CH), 130.97(CH), 139.69(C), 155.8(C), 158.29(C), 162.56(C), 173.99(C). MS:* m/z* = 345 [M]^+^; Anal. Calcd for C_17_H_13_F_2_N_3_OS: C, 59.12; H, 3.79; N, 12.17. Found: C, 58.96; H, 3.67; N, 12.06%.

#### 4.1.7. 4-(2-Chlorophenyl)-5-(6-fluorochroman-2-yl)-4*H*-1, 2, 4-triazole-3-thiol **(4g)**

Yield 76%, mp 192–194°C; IR (DRS): 3077(Ar, C–H str.), 2978(C–H str.), 2563(–SH str.), 1563(–C=C–, str.), 1464(H–C–H bend), 1310(C–O str.), 870(C–Cl str.) cm^−1^; ^1^H NMR (400 MHz, CDCl_3_): *δ* ppm 2.34(s, 6H, 3CH, 3CH), 2.21–2.46(m, 2H, 2CH), 2.75–2.85(m, 2H, 2CH), 4.90–4.97(dd, *J* = 12.6 Hz, 6 Hz, 1H, CH), 6.72–6.95(m, 3H, ArH), 7.39–7.50(m, 3H, ArH), 13.05(s, 1H, SH). ^13^C NMR (100 MHz, CDCl_3_): *δ* ppm 26.27(CH_2_), 28.87(CH_2_), 85.3(CH), 108.52(CH), 112.98(CH), 116.81(CH), 127.83(CH), 126.39(C), 128.04(CH), 129.2(C), 131.85(CH), 132.57(CH), 140.8(C), 142.53(C), 155.8(C), 158.29(C), 175.1(C). MS:* m/z* = 361 [M]^+^; Anal. Calcd C_17_H_13_ClFN_3_OS: C, 56.43; H, 3.62; N, 11.61. Found: C, 55.97; H, 3.55; N, 11.59%.

#### 4.1.8. 4-(3-Chloro-4-fluorophenyl)-5-(6-fluorochroman-2-yl)-4*H*-1,2,4-triazole-3-thiol **(4h)**

Yield 87%, mp 139–141°C; IR (DRS): 3075(Ar, C–H str.), 2553(–SH str.), 1581(–C=C–, str.), 1423(H–C–H bend), 1281(C–O str.), 870(C–Cl str.) cm^−1^; ^1^H NMR (400 MHz, DMSO-d_6_): *δ* ppm 2.34(s, 6H, 3CH, 3CH), 2.21–2.46(m, 2H, 2CH), 2.75–2.85(m, 2H, 2CH), 4.90–4.97(dd, *J* = 12.6 Hz, 6 Hz, 1H, CH), 6.72–6.95(m, 3H, ArH), 7.18–7.79(m, 3H, ArH), 13.05(s, 1H, SH). ^13^C NMR (100 MHz, DMSO-d_6_): *δ* ppm 26.27(CH_2_), 28.87(CH_2_), 85.3(CH), 108.52(CH), 112.98(CH), 116.81(CH), 120.08(CH), 121.91(CH), 124.85(C), 125.71(CH), 129.2(C), 139.69(C), 141.09(C), 155.8(C), 156.41(C), 158.29(C), 173.99(C). MS:* m/z* = 379 [M]^+^; Anal. Calcd for C_17_H_12_ClF_2_N_3_OS: C, 53.76; H, 3.18; N, 11.06. Found: C, 53.69; H, 3.07; N, 10.90%.

#### 4.1.9. 5-(6-Fluorochroman-2-yl)-4-(2-methoxyphenyl)-4*H*-1, 2, 4-triazole-3-thiol **(4i)**

Yield 90%, mp 251–253°C; IR (DRS): 3075(Ar, C–H str.), 2553(–SH str.), 1581(–C=C–, str.), 1423(H–C–H bend), 1281(C–O str.), 870(C–Cl str.), 1080(C–F str.) cm^−1^; ^1^H NMR (400 MHz, DMSO-d_6_): *δ* ppm 2.34(s, 6H, 3CH, 3CH), 2.21–2.46(m, 2H, 2CH), 2.75–2.85(m, 2H, 2CH), 3.83(s, 3H, OCH3), 4.90–4.97(dd, *J* = 12.6 Hz, 6 Hz, 1H, CH), 6.72–6.95(m, 3H, ArH), 6.99–7.51(m, 4H, ArH), 13.05(s, 1H, SH). ^13^C NMR (100 MHz, DMSO-d_6_): *δ* ppm 26.27(CH_2_), 28.87(CH_2_), 56.32(CH_3_), 85.3(CH), 108.52(CH), 112.98(CH), 115.51(CH), 116.81(CH), 120.83(CH), 123.69(CH), 129.2(C), 131.19(CH), 134.73(C), 139.24(C), 155.8(C), 157.76(C), 158.29(C), 173.54(C). MS:* m/z* = 357 [M]^+^; Anal. Calcd for C_18_H_16_FN_3_O_2_S: C, 60.49; H, 4.51; N, 11.76. Found: C, 60.39; H, 4.29; N, 11.37%.

#### 4.1.10. 4-(2, 5-Difluorophenyl)-5-(6-fluorochroman-2-yl)-4*H*-1,2,4-triazole-3-thiol **(4j)**

Yield 75%, mp 229–231°C; IR (DRS): 3061(Ar, C–H str.), 2951 (C–H str.), 2535(–SH str.), 1589(–C=C–, str.), 1462(H–C–H bend), 1520(C–O str.), 1075(C–F str.) cm^−1^; ^1^H NMR (400 MHz, CDCl_3_): *δ* ppm 2.34(s, 6H, 3CH, 3CH), 2.21–2.46(m, 2H, 2CH), 2.75–2.85(m, 2H, 2CH), 3.83(s, 3H, OCH3), 4.90–4.97(dd, *J* = 12.6 Hz, 6 Hz, 1H, CH), 6.72–6.95(m, 3H, ArH), 7.13–7.22(m, 3H, ArH), 13.05(s, 1H, SH). ^13^C NMR (100 MHz, CDCl_3_): 26.27(CH2), 28.87(CH2), 85.3(CH), 108.29(CH), 108.69(CH) 112.98(CH), 116.81(CH), 117.32(CH), 118.79(C), 127.11(CH), 129.2(C), 139.69 (C), 155.8(C), 158.29(C), 159.93(C), 173.54(C). MS:* m/z* = 363 [M]^+^; Anal. Calcd for C_17_H_12_F_3_N_3_O_2_S: C, 56.19; H, 3.33; N, 11.56. Found: C, 56.06; H, 3.14; N, 11.32%.

### 4.2. General Procedure for the Synthesis of Functionalized 4-Thiazolidinones **5a–5j**

An equimolar mixture of 6-fluorochroman-2-carbohydrazide** 2** (0.01 mol) and different aryl aldehydes (0.01 mol) was taken in RBF and to this, thioglycolic acid (mercaptoacetic acid) (0.29 mol) in toluene was added. Then reaction mixture was allowed to reflux in a Dean-Stark assembly with continuous stirring. After completion of the reaction (48 hrs monitoring by TLC), the content was cooled to room temperature and then neutralized with saturated sodium bicarbonate solution. The organic extracts were washed with water and dried over Na_2_SO_4._ The solvent was evaporated* in vacuo* and the resulting crude product was purified by column chromatography to give the analytical pure compounds** 5a–5j**. Column chromatography was carried out in hexane: ethyl acetate solvent system. Pure compound was eluted in 23% ethyl acetate in hexane.

#### 4.2.1. 6-Fluoro-*N*-(2-(4-methoxyphenyl)-4-oxothiazolidine-3-yl) chroman-2-carboxamide **(5a)**

Yield 85%, mp 98–100°C; IR (DRS): 3383(Amide–NH str.), 3081(Ar, C–H str.), 2958(C–H str.), 1714(C=O str.), 1688(amide C=O str.), 1542(–NH bend), 1365(C–F str.), 1278(C–O–C str.) cm^−1^; ^1^H NMR (400 MHz, DMSO-d_6_): *δ* ppm 2.15–2.40(m, 2H, 2CH), 2.75–2.85(m, 2H, 2CH), 3.83(s, 3H, OCH3), 4.69–4.72(d, *J* = 16.0 Hz, 1H, CH), 3.91–3.95(d, *J* = 15.6 Hz, 1H, CH), 4.69–4.72(dd, *J* = 3.6 Hz, 3.2 Hz, 1H, CH), 5.92(s, 1H, CH), 6.72–6.95(m, 3H, ArH), 6.87–7.84(m, 4H, ArH), 8.0(s, 1H, NH), ^13^C NMR (100 MHz, DMSO-d_6_): *δ* ppm 25.25(CH_2_), 26.19(CH_2_), 34.2(CH_2_), 55.33(CH_3_), 70.50(CH), 85.58(CH), 108.7(CH), 112.44(CH), 114.1(CH), 116.43(CH), 126.91(CH), 129.9(C), 133.68(C), 154.35(C), 157.18 (C), 155.8(C), 158.08(C), 165.35(C), 169.36(C). MS:* m/z* = 402 [M]^+^; Anal. Calcd for C_20_H_19_FN_2_O_4_S: C, 59.69; H, 4.76; N, 6.96. Found: C, 59.23; H, 4.61; N, 6.88%.

#### 4.2.2. 6-Fluoro-*N*-(2-(2-nitrophenyl)-4-oxothiazolidine-3-yl) chroman-2-carboxamide **(5b)**

Yield 95%, mp 190–192°C; IR (DRS): 3392(Amide–NH str.), 3205(Ar, C–H str.), 1712(C=O str.), 1678(amide C=O str.), 1523(–NH bend), 1590(N=O str.), 1259(C–O–C str.), 1190(C–F str.) cm^−1^; ^1^H NMR (400 MHz, DMSO-d_6_): *δ* ppm 1.81–2.05(m, 2H, 2CH), 2.61–2.74(m, 2H, 2CH), 3.68–3.72(d, *J* = 16.0 Hz, 1H, CH), 3.91–3.95(d, *J* = 15.6 Hz, 1H, CH), 4.66–4.69(dd, *J* = 3.6 Hz, 3.2 Hz, 1H, CH), 6.12(s, 1H, CH), 6.72–6.79(m, 1H, ArH), 6.84–6.91(m, 2H, ArH), 7.60–7.63(t, 1H, ArH), 7.78–7.87(m, 2H, ArH), 8.04–8.06(d, *J* = 8.0 Hz, 1H, ArH), 10.43–10.46(d, *J* = 11.6 Hz, 1H, NH). ^13^C NMR (100 MHz, DMSO-d_6_): *δ* ppm 25.25(CH_2_), 26.19(CH_2_), 34.2(CH_2_), 68.3(CH), 85.58(CH), 108.7(CH), 114.1(CH), 116.43(CH), 123.91(CH), 125.5(C), 129.87(CH), 129.9(C), 127.85(CH), 132.06(C), 149.87(C), 154.35(C), 157.18 (C), 169.36(C). MS:* m/z* = 417 [M]^+^; Anal. Calcd for C_19_H_16_FN_3_O_5_S: C, 54.67; H, 3.86; N, 10.07. Found: C, 54.58; H, 3.49; N, 9.99%.

#### 4.2.3. 6-Fluoro-*N*-(2-(4-fluorophenyl)-4-oxothiazolidine-3-yl) chroman-2-carboxamide **(5c)**

Yield 89%, mp 106–110°C; IR (DRS): 3487(Amide–NH str.), 3041(Ar, C–H str.), 1710(C=O str.), 1676(amide C=O str.), 1537(–NH bend), 1201(C–O–C str.), 1190(C–F str.) cm^−1^; ^1^H NMR (400 MHz, CDCl_3_): *δ* ppm 1.82–2.02(m, 2H, 2CH), 2.56–2.70(m, 2H, 2CH), 3.59–3.78(m, 2H, 2CH), 4.45–4.58(dd, *J* = 10.52 Hz, 11.52 Hz, 1H, CH), 5.79–5.83(d, *J* = 17.32 Hz, 1H, CH), 6.54–6.72(m, 3H, ArH), 6.86–6.90(t, 1H, ArH), 6.99–7.03(t, 1H, ArH), 7.20–7.22(t, 1H, ArH), 7.33–7.36(t, 1H, ArH), 8.05–8.13(d, *J* = 29.44 Hz, 1H, NH). ^13^C NMR (100 MHz, CDCl_3_): *δ* ppm 25.25(CH_2_), 26.19(CH_2_), 34.2(CH_2_), 70.52(CH), 85.58(CH), 108.7(CH), 113.27(CH), 114.1(CH), 116.43(CH), 127.64(CH), 129.9(C), 136.68(C), 154.35(C), 157.18 (C), 160.07(C), 165.35(C), 169.36(C). MS:* m/z* = 390 [M]^+^; Anal. Calcd for C_19_H_16_F_2_N_2_O_3_S: C, 58.45; H, 4.13; N, 7.18. Found: C, 58.02; H, 4.03; N, 7.10%.

#### 4.2.4. *N-*(2-(3-Chlorophenyl)-4-oxothiazolidine-3-yl)-6-fluorochroman-2-carboxamide **(5d)**

Yield 77%, mp 151–153°C; IR (DRS): 3401(Amide–NH str.), 3200(Ar, C–H str.), 1717(C=O str.), 1645(amide C=O str.), 1550(–NH bend), 1245(C–F str.), 1201(C–O–C str.), 820(C–Cl str.) cm^−1^; ^1^H NMR (400 MHz, DMSO): *δ* ppm 2.15–2.40(m, 2H, 2CH), 2.75–2.85(m, 2H, 2CH), 4.66–4.69(d, *J* = 16.0 Hz, 1H, CH), 3.91–3.95(d, *J* = 15.6 Hz, 1H, CH), 4.69–4.72(dd, *J* = 3.6 Hz, 3.2 Hz, 1H, CH), 5.92(s, 1H, CH), 6.72–6.95(m, 3H, ArH), 7.24–7.43(m, 4H, ArH), 8.0(s, 1H, NH). ^13^C NMR (100 MHz, CDCl_3_): *δ* ppm 25.25(CH_2_), 26.19(CH_2_), 34.2(CH_2_), 71.78(CH), 85.58(CH), 108.7(CH), 114.1(CH), 116.43(CH), 124.5(CH), 124.93(CH), 126.41(CH), 128(CH), 132.25(C), 140.45(C), 145.35(C), 157.18 (C), 165.35(C), 169.36(C). MS:* m/z* = 406 [M]^+^; Anal. Calcd for C_19_H_16_ClFN_2_O_3_S: C, 56.09; H, 3.96; N, 6.89. Found: C, 55.90; H, 3.83; N, 6.83%.

#### 4.2.5. *N*-(2-(4-Bromophenyl)-4-oxothiazolidine-3-yl)-6-fluorochroman-2-carboxamide **(5e)**

Yield 90%, mp 118–120°C; IR (DRS): 3452(Amide–NH str.), 3000(Ar, C–H str.), 1725(C=O str.), 1640(amide C=O str.), 1545(–NH bend), 1345(C–F str.), 1251(C–O–C str.), 520(C–Br str.) cm^−1^; ^1^H NMR (400 MHz, DMSO-d_6_): *δ* ppm 2.15–2.40(m, 2H, 2CH), 2.75–2.85(m, 2H, 2CH), 4.63–4.66(d, *J* = 16.0 Hz, 1H, CH), 3.91–3.95(d, *J* = 15.6 Hz, 1H, CH), 4.63–4.66(dd, *J* = 3.6 Hz, 3.2 Hz, 1H, CH), 5.92(s, 1H, CH), 6.72–6.95(m, 3H, ArH), 7.12–7.85(m, 4H, ArH), 8.0(s, 1H, NH). ^13^C NMR (100 MHz, DMSO-d_6_): *δ* ppm 25.25(CH_2_), 26.19(CH_2_), 34.2(CH_2_), 70.50(CH), 85.58(CH), 108.7(CH), 114.1(CH), 116.43(CH), 120.41(C), 126.7(CH), 129.36(CH), 129.9(C), 138.93(C), 154.35(C), 157.18 (C), 165.35(C), 169.36(C). MS:* m/z* = 452 [M+1]^+^; Anal. Calcd for C_19_H_16_BrFN_2_O_3_S: C, 50.56; H, 3.57; N, 6.21. Found: C, 50.45; H, 3.28; N, 6.11%.

#### 4.2.6. 6-Fluoro-*N*-(2-(4-hydroxyphenyl)-4-oxothiazolidine-3-yl) chroman-2-carboxamide **(5f)**

Yield 73%, mp 132–134°C; IR (DRS): 3452(Amide–NH str.), (–OH, broad), 3011(Ar, C–H str.), 1728(C=O str.), 1645(amide C=O str.), 1545(–NH bend), 1352(C–F str.), 1278(C–O–C str.) cm^−1^; ^1^H NMR (400 MHz, DMSO-d_6_): *δ* ppm 2.15–2.40(m, 2H, 2CH), 2.75–2.85(m, 2H, 2CH), 4.63–4.66(d, *J* = 16.0 Hz, 1H, CH), 3.91–3.95(d, *J* = 15.6 Hz, 1H, CH), 4.63–4.66(dd, *J* = 3.6 Hz, 3.2 Hz, 1H, CH), 5.35(s, 1H, OH), 5.92(s, 1H, CH), 6.72–6.95(m, 3H, ArH), 7.14–7.86(m, 4H, ArH), 8.0(s, 1H, NH). ^13^C NMR (100 MHz, DMSO-d_6_): *δ* ppm 25.25(CH_2_), 26.19(CH_2_), 34.2(CH_2_), 55.33(CH_3_), 70.50(CH), 85.58(CH), 108.7(CH), 114.1(CH), 116.43(CH), 116.68(C), 125.81(CH), 129.9(C), 132.68(C), 154.35(C), 157.46 (C), 157.18(C), 165.35(C), 169.36(C). MS:* m/z* = 388 [M]^+^; Anal. Calcd for C_19_H_17_FN_2_O_4_S: C, 58.75; H, 4.41; N, 7.21. Found: C, 58.56; H, 4.34; N, 7.06%.

#### 4.2.7. 6-Fluoro-*N*-(4-oxo-2-(*p*-tolyl) thiazolidin-3-yl) chroman-2-carboxamide **(5g)**

Yield 84%, mp 89–91°C; IR (DRS): 3328(Amide–NH str.), 2998(Ar, C–H str.), 2900(C–H str.), 1741(C=O str.), 1652(amide C=O str.), 1548(–NH bend), 1371(C–F str.), 1300(C–O–C str.) cm^−1^; ^1^H NMR (400 MHz, DMSO-d_6_): *δ* ppm 2.15–2.32(m, 2H, 2CH), 2.34(s, 3H, 3CH), 2.75–2.85(m, 2H, 2CH), 4.63–4.66(d, *J* = 16.0 Hz, 1H, CH), 3.91–3.95(d, *J* = 15.6 Hz, 1H, CH), 4.63–4.66(dd, *J* = 3.6 Hz, 3.2 Hz, 1H, CH), 5.92(s, 1H, CH), 6.72–6.95(m, 3H, ArH), 7.11(m, 4H, ArH), 8.0(s, 1H, NH). ^13^C NMR (100 MHz, DMSO-d_6_): *δ* ppm 21.03(CH_3_), 25.25(CH_2_), 26.19(CH_2_), 34.2(CH_2_), 70.50(CH), 85.58(CH), 108.7(CH), 114.1(CH), 116.43(CH), 126.73(CH), 127.98(CH), 129.9(C), 136.48(C), 136.52(C), 154.35(C), 157.18(C), 165.35(C), 169.36(C). MS:* m/z* = 386 [M]^+^; Anal. Calcd C_20_H_19_FN_2_O_3_S: C, 62.16; H, 4.96; N, 7.25. Found: C, 62.07; H, 4.55; N, 7.09%.

#### 4.2.8. *N*-(2-(2-Chlorophenyl)-4-oxothiazolidine-3-yl)-6-fluorochroman-2-carboxamide **(5h)**

Yield 71%, mp 158–160°C; IR (DRS): 3412(Amide–NH str.), 3212(Ar, C–H str.), 1725(C=O str.), 1685(amide C=O str.), 1558(–NH bend), 1251(C–F str.), 1228(C–O–C str.), 800(C–Cl str.), 770(o-di substituted) cm^−1^; ^1^H NMR (400 MHz, DMSO): *δ* ppm 2.15–2.32(m, 2H, 2CH), 2.75–2.85(m, 2H, 2CH), 4.63–4.66(d, *J* = 16.0 Hz, 1H, CH), 3.91–3.95(d, *J* = 15.6 Hz, 1H, CH), 4.63–4.66(dd, *J* = 3.6 Hz, 3.2 Hz, 1H, CH), 5.92(s, 1H, CH), 6.72–6.95(m, 3H, ArH), 7.17–7.65(m, 4H, ArH), 8.0(s, 1H, NH). ^13^C NMR (100 MHz, CDCl_3_): *δ* ppm 25.25(CH_2_), 26.19(CH_2_), 34.2(CH_2_), 67.50(CH), 85.58(CH), 108.7(CH), 114.1(CH), 116.43(CH), 125.18(CH), 125.89(CH), 127.02(CH), 127.26(CH), 129.9(C), 131.68(C), 133.39(C), 154.35(C), 157.18 (C), 165.35(C), 169.36(C). MS:* m/z* = 406 [M]^+^; Anal. Calcd for C_19_H_16_ClFN_2_O_3_S: C, 56.09; H, 3.96; N, 6.89. Found: C, 55.93; H, 3.77; N, 6.84%.

#### 4.2.9. 6-Fluoro-*N*-(2-(4-nitrophenyl)-4-oxothiazolidine-3-yl) chroman-2-carboxamide **(5i)**

Yield 79%, mp 207–209°C; IR (DRS): 3398(Amide–NH str.), 3212(Ar, C–H str.), 1728(C=O str.), 1688(amide C=O str.), 1543(–NH bend), 1595(N=O str.), 1272(C–O–C str.), 1290(C–F str.) 790(p-di substituted) cm^−1^; ^1^H NMR (400 MHz, CDCl_3_): *δ* ppm 2.15–2.32(m, 2H, 2CH), 2.75–2.85(m, 2H, 2CH), 4.63–4.66(d, *J* = 16.0 Hz, 1H, CH), 3.91–3.95(d, *J* = 15.6 Hz, 1H, CH), 4.63–4.66(dd, *J* = 3.6 Hz, 3.2 Hz, 1H, CH), 5.92(s, 1H, CH), 6.72–6.95(m, 3H, ArH), 7.49–8.14(m, 4H, ArH), 8.0(s, 1H, NH). ^13^C NMR (100 MHz, CDCl_3_): *δ* ppm 25.25(CH_2_), 26.19(CH_2_), 34.2(CH_2_), 70.52(CH), 85.58(CH), 108.7(CH), 114.1(CH), 116.43(CH), 122.91(CH), 126.32(CH), 129.9(C), 144.08(C), 145.35(C), 154.35 (C), 157.18(C), 165.35(C), 169.36(C). MS:* m/z* = 417 [M]^+^; Anal. Calcd for C_17_H_14_FN_3_O_2_: C, 54.67; H, 3.86; N, 10.07. Found: C, 54.39; H, 3.79; N, 9.89%.

#### 4.2.10. *N*-(2-(3-Bromophenyl)-4-oxothiazolidine-3-yl)-6-fluorochroman-2-carboxamide **(5j)**

Yield 88%, mp 166–168°C; IR (DRS): 3482(Amide–NH str.), 3110(Ar, C–H str.), 1745(C=O str.), 1590(amide C=O str.), 1485(–NH bend), 1354(C–F str.), 1281(C–O–C str.), 680(m-di substituted), 520(C–Br str.) cm^−1^; ^1^H NMR (400 MHz, CDCl_3_): *δ* ppm 2.15–2.32(m, 2H, 2CH), 2.75–2.85(m, 2H, 2CH), 4.63–4.66(d, *J* = 16.0 Hz, 1H, CH), 3.91–3.95(d, *J* = 15.6 Hz, 1H, CH), 4.63–4.66(dd, *J* = 3.6 Hz, 3.2 Hz, 1H, CH), 5.92(s, 1H, CH), 6.72–6.95(m, 3H, ArH), 7.22–7.41(m, 4H, ArH), 8.0(s, 1H, NH). ^13^C NMR (100 MHz, CDCl_3_): *δ* ppm 25.25(CH_2_), 26.19(CH_2_), 34.2(CH_2_), 70.32(CH), 85.58(CH), 108.7(CH), 114.1(CH), 116.43(CH), 120.91(C), 127.55(CH), 127.95(CH), 128.17(CH), 128.74(CH), 129.9(C), 140.65(C), 154.35(C), 157.18 (C), 165.35(C), 169.36(C).MS:* m/z* = 452 [M+1] ^+^; Anal. Calcd for C_19_H_16_FN_3_O_5_S: C, 50.56; H, 3.57; N, 6.21. Found: C, 50.17; H, 3.14; N, 6.12%.

## Figures and Tables

**Table 1:** Physical data for **4a**–**4j**.

Entry	Substitution R	Yield (%)
**4a**	4-CH_3_ phenyl	89
**4b**	3-Cl phenyl	86
**4c**	4-F phenyl	78
**4d**	2,5-Dimethyl phenyl	95
**4e**	3,4-Dimethyl phenyl	84
**4f**	2-F phenyl	79
**4g**	2-Cl phenyl	76
**4h**	3-Cl-4-F phenyl	87
**4i**	2-OCH_3 _phenyl	90
**4j**	2,5-Difluoro phenyl	75

**Table 2:** Physical data for **5a**–**5j**.

Entry	Substitution R	Yield (%)
**5a**	4-OCH_3_ phenyl	85
**5b**	2-NO_2_ phenyl	95
**5c**	4-F phenyl	89
**5d**	3-Cl phenyl	77
**5e**	4-Br phenyl	90
**5f**	4-OH phenyl	73
**5g**	4-CH_3_ phenyl	84
**5h**	2-Cl phenyl	71
**5i**	4-NO_2_ phenyl	79
**5j**	3-Br phenyl	88
